# Prioritizing Context-Dependent Cancer Gene Signatures in Networks

**DOI:** 10.3390/cancers17010136

**Published:** 2025-01-03

**Authors:** Enrico Capobianco, Thomas S. Lisse, Sandra Rieger

**Affiliations:** 1 NUDDHA LLC, Lake Worth, FL 33463, USA; 2 Avantyx Pharmaceuticals, Miami, FL 33136, USA; tlisse@avantyxpharma.com (T.S.L.); srieger@miami.edu (S.R.); 3 Department of Biology, University of Miami, Coral Gables, FL 33146, USA; 4 Sylvester Comprehensive Cancer Center, Miller School of Medicine, University of Miami, Miami, FL 33136, USA

**Keywords:** cancer, osteosarcoma metastasis, gene signatures, interactome networks

## Abstract

There are numerous ways of portraying cancer complexity based on combining multiple types of data. A common approach involves developing signatures from gene expression profiles to highlight a few key reproducible features that provide insight into cancer risk, progression, or recurrence. Normally, a selection of such features is made through relevance or significance, given a reference context. In the case of highly metastatic cancers, numerous gene signatures have been published with varying levels of validation. Then, integrating the signatures could potentially lead to a more comprehensive view of the connection between cancer and its phenotypes by covering annotations not fully explored in individual studies. This broader understanding of disease phenotypes would improve the predictive accuracy of statistical models used to identify meaningful associations. We present an example of this approach by reconciling a great number of published signatures into meta-signatures relevant to Osteosarcoma (OS) metastasis. We generate a well-annotated and interpretable interactome network from integrated OS gene expression signatures and identify key nodes that regulate essential aspects of metastasis. While the connected signatures link diverse prognostic measurements for OS, the proposed approach is applicable to any type of cancer.

## Simple Summary

Cancer signatures are very popular and support inference when reproducible and generalizable. However, every approach derives them differently, often in a context-specific way. Depending on the available experiments and data, computational methods used to obtain the signatures may vary. A few relevant questions motivated this research, one being the significance of collating signatures in terms of the outcomes, another being the synergistic meaning of a meta or super signature, and finally, whether some effective operational strategy exists to reduce the complexity of a signature set.

## 1. Cancer Gene Signatures

Recently, a retrospective work over the past 25 years of cancer research on gene signatures has been published [1]. The remark that there are limited overlap and actual inconsistencies among only partially reproducible gene signatures across different cohorts is factual and has also inspired our work. Here, the diverse experimental designs, sequencing technologies, and data analysis algorithms producing the signatures are assumed as given, and selected examples from the recent literature with reference to a specific cancer (i.e., osteosarcoma) were considered to focus on integration strategies allowing robust gene signature identification.

Gene expression profiling is commonly used to identify prognostic and predictive cancer signatures, ideally complementing histopathological and clinical assessments. Generally, gene expression signatures (GES) are derived from various datasets, but when the profile heterogeneity refers to the same context, overlapping rather than distinct measurements are found. Biologically, these overlaps may occur within the same cancer or across different cancers, as they often capture shared biological mechanisms present under various conditions. Clinically, all such signatures need to be validated in independent datasets or patient cohorts to ensure clear interpretability and broad applicability.

The informative value and significance of GES can, in principle, be enhanced by integrating additional heterogeneous data, such as electronic health records, clinical markers, and imaging. While GES profiles help explain specific tumor phenotypes or hallmarks, incorporating other omics data—such as mutational profiles, single-cell information, non-coding RNA, methylation, and imaging—can provide further insight. However, this multimodal approach introduces new complexity with regard to the required validation beyond testing GES reproducibility alone. While traditional factors, including selected cohorts, experimental data, in vitro/in vivo systems, and pre-clinical models (e.g., cell lines, patient-derived xenografts (PDX), organoids, etc.) remain essential for conducting inference, computational algorithms now play a main role as they integrate diverse data at the systems’ level, thereby enhancing predictive power. Consequently, integrative, multimodal data science is expected to improve inference results by capturing a ‘wider phenotype spectrum’ through the combination of multiple measurements [2].

Assuming that consistent signatures better support cancer inference as they potentially enhance reproducibility and generalizability, the applicability of any approach is often context-specific rather than universal. Also, various challenges arise with computational methods (such as Machine/Deep Learning (ML/DL), AI, Internet of Medical Things (IoMT), etc.) at different levels:

- **Data:** How significantly does the integration of GES impact the outcomes? The selected profiles used in the integration process can introduce biases that affect the results.
- **Model:** How is significance determined when dealing with meta- or super-signatures? Additionally, how does the chosen strategy for GES integration influence the measurement of significance?
- **Cancer:** Does reduced complexity of the signature landscape improve reproducibility and generalizability across different cancer contexts and/or types?

These considerations substantiate the challenge we face in standardizing and operationalizing the results of cancer inference approaches across the mix of experimental and computational frameworks generating them.

## 2. Osteosarcoma Context

We present an application focused on osteosarcoma (OS) metastasis-related GES. Our literature survey is rich in recent examples undergoing multiple validations (about 5 years of publications coverage). As with other malignancies, OS research has focussed on generating gene signatures that provide valuable diagnostic, therapeutic, and prognostic insight. However, it is challenging to know whether these signatures relate to one another in their results, thus becoming more informative together than in separate assessments. This is a crucial aspect, as the literature shows a proliferation in both the number and the diversity of cancer signatures. Therefore, once a certain context is given, determining whether these signatures can be reconciled and/or connected becomes important for establishing significant and accurate inference.

OS is the most frequent primary malignant bone tumor, with numerous pathophysiological processes potentially underpinning disease progression and metastasis. Driver mutations acting as effective treatment targets and predictive biomarkers of potential treatment effectiveness are currently lacking [3]. Our review of OS-related GES signatures identifying new patient stratifications through risk factors, biological pathways, and processes revealed generalizability and translatability as the most common challenges [4,5]. In other terms, in most studies, each signature presents a value that is highly contextual but not easy to replicate [6,7,8,9]. Based on the available literature, we prioritized GES specific to OS metastasis, our reference context, to build a compendium of selected significant signatures. Metastasis-related categories indicating directly or indirectly relevant ‘meta-signatures’ included prognostic [10,11,12,13,14,15,16,17,18,19], metabolism [20,21,22,23,24], apoptosis [25,26,27], senescence [28,29], epithelial-to-mesenchymal transition (EMT) [30,31,32], hypoxia [33,34,35], autophagy [36,37,38], TME [39,40,41,42,43,44,45,46,47,48,49,50,51,52,53,54,55,56], metastasis [57,58,59,60,61,62,63,64,65], and immune [66,67,68,69,70,71,72,73,74,75,76,77,78,79,80,81,82,83], as detailed in Table 1. A few standard sources (GEO, TCGA, TARGET etc.) were explored, and the strategies for identifying the signatures leveraged statistical methods (LASSO, etc.), ML and DL, complex networks, with risk models validated through various approaches.

**Figure 1:**
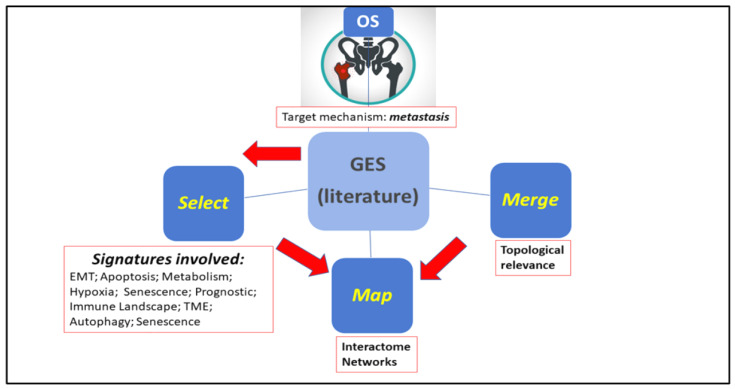
Approach. Steps include literature-based coverage of target metastasis-related signatures, assessment of topological relevance within interactome networks, and integration of signatures. Graphical sketch from PowerPoint artwork.

The strategy of our approach is outlined in Figure 1. With ‘metastasis’ as the target mechanism, the meta-signatures were obtained by merging individual GES, then mapped onto interactome networks outsourced from common repositories, i.e., Innate.DB, STRING.DB, IntAct.DB, HuRI.DB. Specifically, Innate.DB (https://www.innatedb.com/ (accessed on 2023)) covers genes, proteins, experimentally verified interactions, and signaling pathways involved in the innate immunity interactome (>18,780 known interactions and pathways integrated from public databases and manually curated data). STRING.DB (https://string-db.org/ (accessed on 2023) contains information from multiple sources (experimental data, computational predictions, etc.). IntAct.Db (https://www.ebi.ac.uk/intact/home (accessed on 2023)) provides evidence of molecular interactions, while HuRI.DB (http://www.interactome-atlas.org/ (accessed on 2023)) is linked to the Human Reference Interactome Mapping Project and reports proteome-scale PPI datasets. These tools can be queried separately or via dedicated portals (see, for instance, https://www.networkanalyst.ca/ (accessed on 2023)).

## 3. Qualitative and Quantitative Assessments

**Figure 2:**
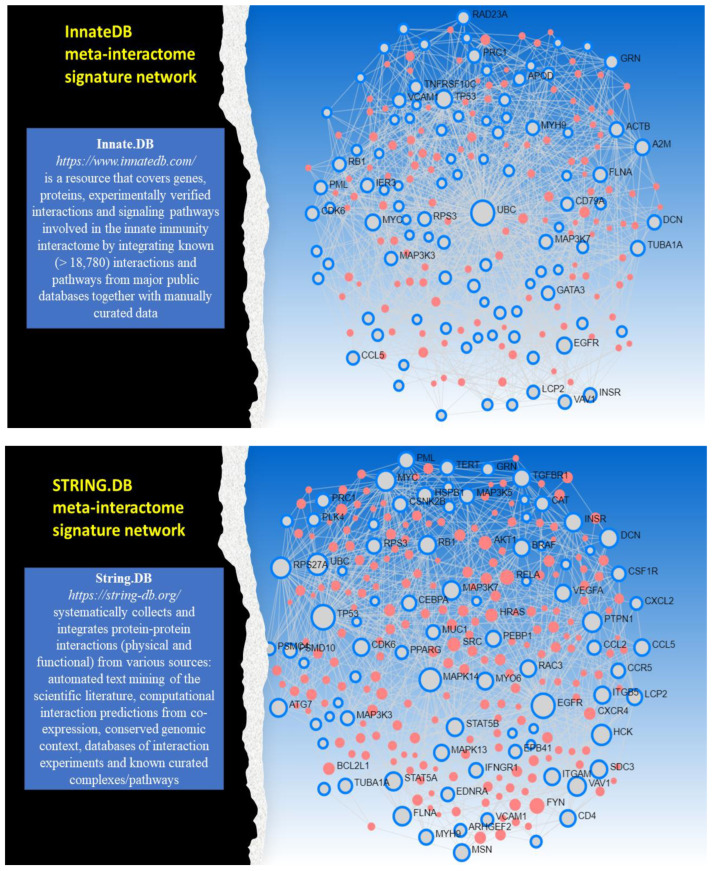
Meta-interactome signature networks. Innate.DB and STRING.DB as examples of network sources. The blue-circled nodes are network backbone components of the meta-signature; the red nodes are associated interactors. The plots show protein interactions putting signature components in relationships with biologically associated nodes.

We performed both quantitative and qualitative assessments. To reduce the redundancy of the interactions and improve the interpretability of the network bio-annotations, the configurations were pruned to a minimal size. This reduction preserves most connectivity and prioritizes the links between the most salient nodes according to common topological properties. The confidence of the interactions was a retained parameter in STRING.DB to determine the configuration size based on a mix of validated hypotheses and experimental evidence annotated within the system. Further refinement at the topological level was obtained by computing two general measures, degree (node connectivity, NCO) and betweenness (node centrality, NCE), and keeping only nodes with NCO and NCE values above fixed thresholds. These were the nodes forming the backbone of the so-called ‘meta-signatures’ associated with the meta-interactome networks (see Figure 2). The biological interpretation was supported by standard bio-annotations obtained from *GO:BP* (biological processes) and pathways (source: *REACTOME*).

### 3.1. Interactomes Commonality and Diversity

Forty percent of the top ten topologically significant nodes are shared between the two primary sources, including *MYC, EGFR, UBC*, and *TP53* (Table 2). Notably, the first entry in the Innate.DB list identifies UBC as topologically dominant across both the considered topological measures, more so than in STRING.DB. As a reminder, dysregulated *UBC* has garnered attention in OS tumorigenesis and treatment, exemplified by a study identifying a ubiquitin-related prognostic gene signature that predicts the immune landscape and OS molecular subtypes [84]. Also of relevance is the ranking of *MYC*, recognized as identifying a distinct OS subtype [85].

**Figure 3:**
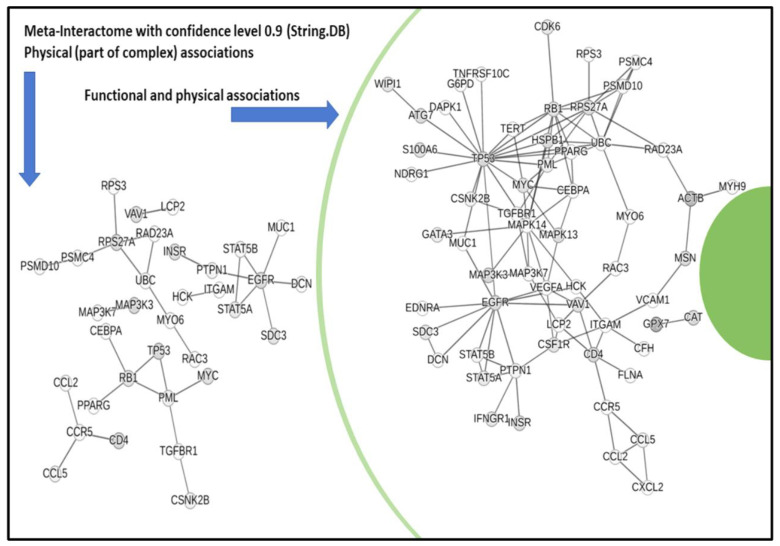
Physical associations then expanded in functional–physical view. The map has been significantly reduced according to the most stringent confidence level of the interactions (level = 0.9).

**Figure 4:**
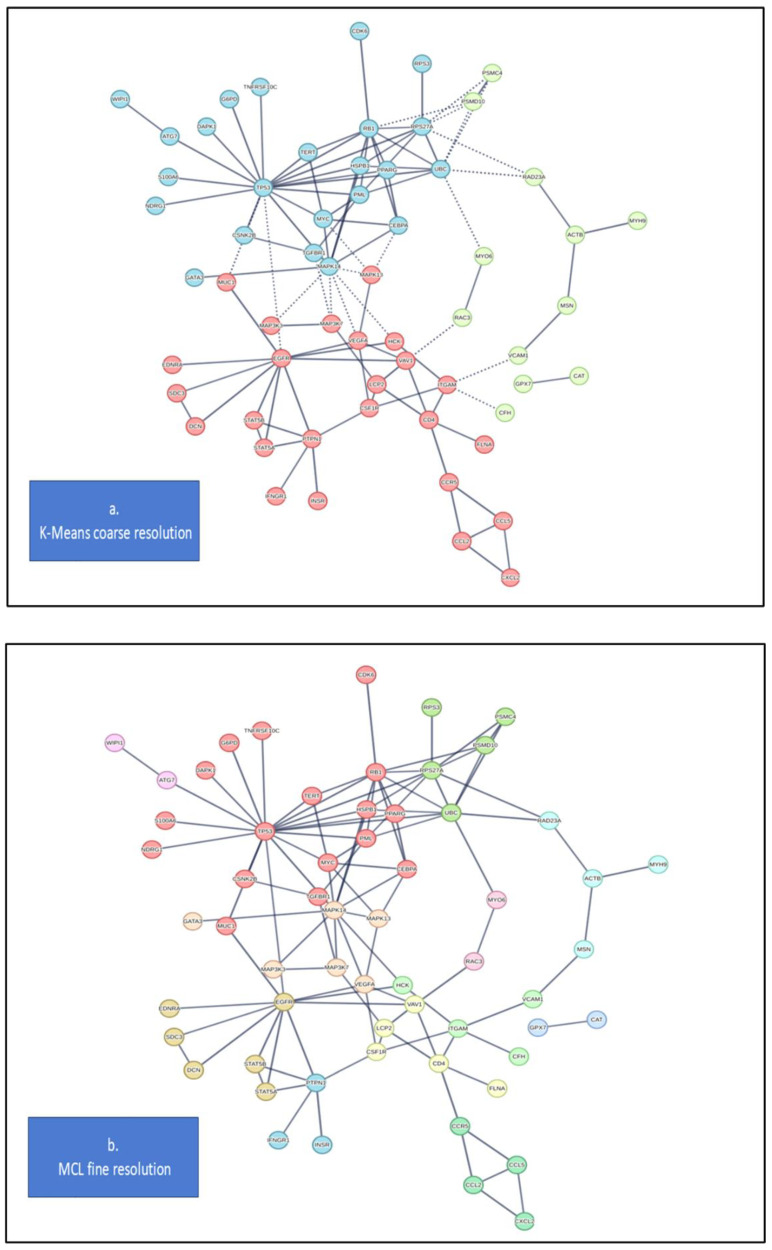
STRING.DB clustering of the functional–physical meta-interactome network. Two standard methods were employed: (**a**) K-Means: 3 clusters are separated by color and serve as a coarse reference for associations (see, for instance, the *EGFR* and *TP53* central nodes). (**b**) Markov Cluster Algorithm (MCL): more groups appear as a result of a finer resolution. As a note, these two clustering methods are complementary, being K-Means based on an arbitrary definition of the number of groups, while MCL is implicitly controlled by an inflation parameter.

In terms of bio-annotations (source REACTOME), the top pathway enrichment terms (Table 3) include the immune system, signaling by interleukins, platelet activation, innate immune system, adaptive immune system, cytokine signaling, and these terms appear largely shared between the two systems. The top-ranked terms appear similar despite noticeable differences in system size and hits. GO:BP (biological processes) annotations (Supplementary File S1) show terms like regulation of protein metabolic processes and intracellular protein kinase cascade, which are similarly ranked. Noteworthy among the top annotated terms are two fingerprints, one of which is apoptosis in Innate.DB and one of phosphorylation in STRING.DB. Regarding phosphorylation and the altered metabolism in OS, the role of metabolic reprogramming relevant to cancer progression is especially linked to oxidative phosphorylation. Annotations were also obtained from the networks with multiple connectivity patterns retrieved by mapping and integrating the signatures at the network scale. Both physical and functional networks were visualized (Figure 3), with validating information provided about involved protein complexes. A physical backbone presents three main hubs centered on *EGFR; UBC*; and the combined *PML, TP53, RB1,* and *MYC* group. To better delineate associations, clustering was employed at both coarse (K-Means method, Figure 4a) and fine (Markov Clustering or MCL, Figure 4b) levels. Interpretation of these results is presented in the following section (with annotations retrieved from the STRING.DB repository).

Clustering effects of *EGFR* are also seen with groups involved in stress response and apoptosis, including the following:

(a) *MAP3K3*, which directly regulates the stress-activated protein kinase (*SAPK*) and extracellular signal-regulated protein kinase (*ERK*) pathways by activating *SEK* and *MEK1/2,* respectively, and (b) *MAP3K7*, a member of the serine/threonine protein kinase family that mediates signaling transduction and controls a variety of cell functions including transcription regulation and apoptosis.

Notably, *EGFR* also clusters with another group regulating cell cycle, cell growth, tumor suppression, OS, cellular stress response, apoptosis, senescence, transcription, metabolism, and including the transcription factor *CEBPA* (this contains a basic leucine zipper (*bZIP*) domain and recognizes the CCAAT motif in the promoters of its target genes (modulating gene expression in cell cycle regulation).

Other key network components, partly established in cancer studies, include the following:

(a) *RB1*, a tumor suppressor and negative cell cycle regulator that stabilizes constitutive heterochromatin to maintain the chromatin structure (note that defects in this gene are a cause of various childhood cancers);
(b) *TP53*, encoding a tumor suppressor that responds to cellular stress by regulating gene expression and inducing cell cycle arrest, apoptosis, senescence, DNA repair, and metabolic changes;
(c) *PML*, a phosphoprotein localized to nuclear bodies and functioning both as a TF and as a tumor suppressor, regulating the p53 response to oncogenic signals;
(d) *MYC*, a major player in cell cycle progression, apoptosis, and cellular transformation (its gene amplification is frequently observed in numerous human cancers);
(e) *PPARG*, encoding a nuclear receptor that belongs to the peroxisome proliferator-activated receptor (PPAR) subfamily and regulating the transcription of genes implicated in disease pathology, including cancer.

Finally, the ubiquitin-related group, which, through ubiquitination, indices protein degradation and plays a critical role in processes like DNA repair, cell cycle regulation, kinase modification, endocytosis, and regulation of other cell signaling pathways. Several ubiquitin-linked nodes were identified:

(a) *RPS3*, a ribosomal protein belonging to the S3P family;
(b) *RPS27A*, encoding a fusion protein with ubiquitin at the N terminus and ribosomal protein S27a at the C terminus;
(c) *PSMD10*, a regulatory component of the 26S proteasome complex, which is essential for ubiquitin-dependent protein degradation (whose aberrant expression may contribute to tumorigenesis). Other nodes include the following:
(d) *UBC*, a gene encoding protein the precursor protein, polyubiquitin, which, depending on ubiquitin conjugation sites, exerts various intracellular effects);
(e) *RAC3*, a GTPase from the RAS superfamily of small GTP-binding proteins that regulate diverse cellular events, including the control of cell growth, cytoskeletal reorganization, and protein kinase activation.

### 3.2. Network Inference

From observations at the network scale, some groups have emerged as interconnected at the physical interaction level. The first group is centered on a T-cell pair, *VAV1-LCP2*. *VAV* proteins activate pathways leading to actin cytoskeletal rearrangements and transcriptional changes, playing critical roles in hematopoiesis and in the development and activation of T-cells and B-cells. LCP2, an encoded adapter protein, functions as a substrate in the T cell antigen receptor (TCR)-activated protein tyrosine kinase pathway, thereby contributing to TCR-mediated intracellular signal transduction. The second group includes members of the chemokine superfamily of secreted proteins involved in immunoregulatory and inflammatory processes, including *CCL5* and *CCR5* (encoding a protein expressed by T cells and macrophages) together with *CCL2* and *CD4* (associated with T lymphocytes). Our analysis provides further relationships by adding functional links between the above groups. An example is provided by *VEGFA*, a member of the PDGF/VEGF growth factor family. *VEGFA* induces the proliferation and migration of vascular endothelial cells and is essential for both physiological and pathological angiogenesis. This gene is upregulated in various tumors, and its expression is often correlated with tumor stage and progression. Group associations are further clarified by clustering and networks (with annotations supported by GeneCards—Human Genes Database (https://www.genecards.org (accessed on 2023)).

These two groups (T-cell and chemokine) are naturally clustered in Figure 4, with the T-cell group additionally connected with a third group centered on *EGFR* and highlighting tumorigenesis, proliferation, apoptosis, signaling, angiogenesis. *EGFR* is widely recognized in cancer as a cell surface protein that induces cell proliferation. This transmembrane glycoprotein belongs to the protein kinase superfamily and is a receptor for other members of the epidermal growth factor family. This third group includes a number of proteins:

(a) *MUC1,* which encodes a membrane-bound protein that belongs to the mucin family and is primarily expressed on the apical surface of epithelial cells and plays a role in cancer. *MUC1* is often overexpressed and abnormally glycosylated in many cancers, including breast, pancreatic, and lung cancer. In cancer cells, *MUC1* supports tumor progression by promoting resistance to apoptosis. Its interactions with signaling pathways, like PI3K/Akt, further enhance tumorigenic functions, supporting cell growth and survival. *MUC1* also influences the tumor microenvironment by interacting with factors that promote angiogenesis, contributing to detachment from the primary site and facilitating migration and invasion.
(b) *DCN* (decorin) encodes a member of the small leucine-rich proteoglycan family of proteins with a tumor suppression function, i.e., stimulatory effect on autophagy and inflammation and inhibitory effect on angiogenesis and tumorigenesis, due to binding to multiple cell surface receptors.
(c) *STAT5A* is a member of the STAT family of transcription factors (TFs) and a protein whose activation is essential for tumorigenesis.
(d) *STAT5B* is involved in various biological processes, such as TCR (T cell receptor) signaling apoptosis.
(e) *PTPN1* encodes a protein that is the founding member of the protein tyrosine phosphatase (PTP) family. These proteins are known signaling molecules that regulate a variety of cellular processes, including cell growth, differentiation, mitotic cycle, and oncogenic transformation.

### 3.3. Network Perturbation to Investigate Latent Influences and Regulators

To explore the latent influences potentially induced by the presence of recently identified key OS regulators and assess the changes in the meta-signature landscape, network perturbation was applied by mapping new regulative nodes onto the meta-interactome. For instance, three TFs showed promise in recent studies, thus warranting further investigation. *SNAI2* (known as transrepressor SLUG) is a master regulator (together with *SNAI1*) in the epithelial-to-mesenchymal transition (EMT) of cancer cells. EMT involves epithelial cells transitioning to a mesenchymal phenotype, gaining properties like motility and invasiveness. Although TFs, signaling pathways, and regulatory networks involved in EMT are complex and not yet fully understood, *SNAI1/2* drives the transcription of pro-mesenchymal genes while repressing epithelial genes. The role of *SNAI2* in novel OS-inhibiting mechanisms involving Vitamin D has recently been highlighted [86]. In this study, the impact of vitamin D and its receptor (VDR) was assessed on the NMD-ROS-EMT signaling axis using both in in vitro and in vivo OS animal models. The active vitamin D derivative 1,25(OH)2D inhibited EMT in OS subtypes through VDR signaling. Specifically, ligand-bound VDR directly downregulated the EMT inducer *SNAI2*, differentiating high metastatic from low metastatic subtypes showing 1,25(OH)2D sensitivity.

**Figure 5:**
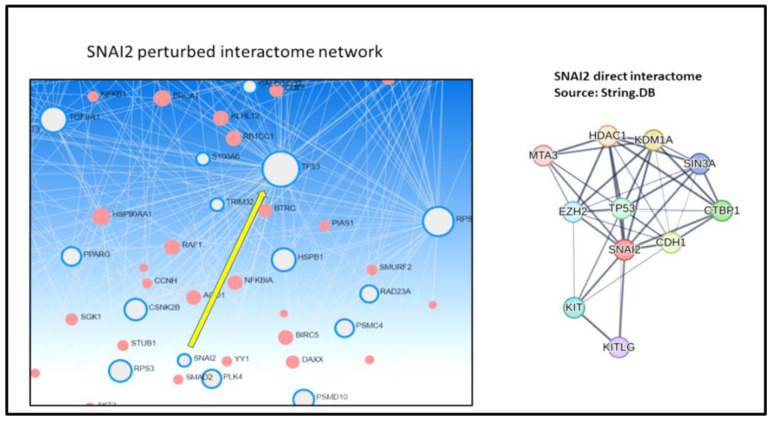
Perturbed meta-interactome network. From STRIG.DB, the *SNAI2* perturbation is shown with the node mapped onto the network (**left**), and the specific *SNAIL2* interactome is visualized (**right**). The induced *SNAI2* interactome associations suggest potential network influence.

In Figure 5 (left panel), an OS-perturbed map shows *SNAI2* within the meta-interactome, connected to *TP53*. The normally induced *SNAI2* interactome (Figure 5, right panel), as shown in STRING.DB, suggests potential network influence. Further text mining on TFs was performed via TRRUST [87], which contains 8444 TF-target regulatory relationships of 800 human TFs from pubmed articles describing small-scale experimental studies of transcriptional regulations. This analysis revealed shared targets between *SNAI2* and both *TP53* and *PPARG*, also present in the meta-interactome but not directly linked to *SNAI2* (Supplementary File S2). Cross-referencing using EMTOME (http://www.emtome.org/ (accessed on 2023)) [88], a portal for exploring pan-cancer enrichments in the EMT context, indicated significant correlation (FDR < 0.05) between *SNAI2* RNA expression in a few TCGA cohorts, such as breast invasive carcinoma (BRCA), kidney renal clear cell carcinoma (KIRK), stomach adenocarcinoma (STAD) (Supplementary Files S3–S5). Of special interest is that KIRK showed similarities to OS regarding pathway enrichments for platelet activation and TCR/BCR signaling, with platelet activation, in particular, exerting notable network influence due to its connection to the blood circulation environment (Supplementary File S4).

Interestingly, while EMT contributes invasive properties of OS cells and links to the microenvironment, these factors alone may be insufficient for metastasis when generalizing to KIRK, as here platelets play a key role through interactions with tumor cells. The consideration of EMT beyond the tissue microenvironment could provide a deeper understanding of the metastatic process [89], aligning with findings that a platelet scoring biomarker significantly determines OS subtypes and influences prognosis [90]. This result is supported by the identification of multiple platelet-related genes distinguishing two subtypes based on immune response: one subtype shows inhibited immune response, while the other has a higher immune cell distribution of immune cells.

**Figure 6:**
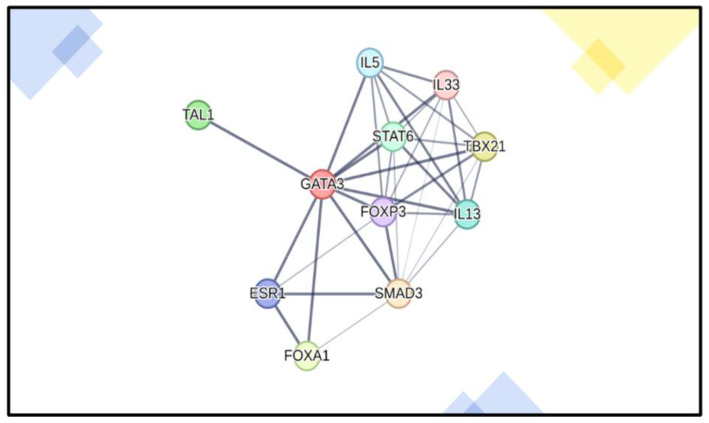
*GATA3* direct interactome (source: STRING.DB). Interactome of anticancer activity genes related to *GATA3*, which is downregulated in OS cells and tissues and whose expression levels depend on factors such as tumor size, metastasis, and suppression of proliferation, migration and invasion.

Our second example is *GATA3*, a TF identified in the metastasis GES (Table 1) and present in both the meta-transcriptome and the meta-interactome backbone (Figure 2, Innate.DB), where it relates to anticancer genes binding (see Figure 6 and Supplementary File S6). In previous work [91], we aimed to identify functional chromosomal regions that may govern anticancer responses and may be coregulated by 1,25(OH)_2_D and oxidative stress using ATACseq (Assay for Transposase-Accessible Chromatin with sequencing) for TF binding motif analysis. Overall, 1,25(OH)_2_D was found to promote chromatin accessibility in MG-63 cancer cells, enhancing the regulatory effects of specific TFs that may play important roles in oxidative stress defense and normal tissue and cellular developmental processes. Analysis using hypergeometric optimization of motif enrichment (HOMER) for TF motif discovery within 1,25(OH)_2_D-sensitive open chromatin revealed that *GATA3* was the most highly associated TF, essential for normal tissue development, and frequently mutated in breast cancers. An important observation is that *GATA3* is downregulated in OS cells and tissues, with expression levels associated with tumor size, metastasis, and suppression of proliferation, migration, and invasion in OS. EMT regulation was also examined using the TF *SLUG* (*SNAI2*), where *GATA3* was observed to transcriptionally inhibit *SLUG* [92], although this association was not referenced in the TRRUST db (Supplementary File S6).

Our third example focuses on *EGFR*, a key node driving OS clustering at the meta-interactome level and a relevant marker for cell proliferation and resistance. *EGFR* is associated with various wound-healing functions, including the proliferation and migration of epidermal keratinocytes, fibroblasts, and endothelial cells in both healthy and pathological contexts. RNA-Seq experiments have demonstrated that ROS oxidization enhances *EGFR* receptor signaling, thereby promoting migration [93]. Notably, while OS resistance to chemotherapy remains an active area of research, the role of *EGFR* signaling and functional contributions, such as hyperactivation, to tumor biology and chemoresistance in OS are still not fully understood. In our study, *EGFR* emerges within meta-signatures linked to autophagy and hypoxia (Table 1), patterns observed in the literature for other cancers, such as lung cancer [94]. *EGFR* is also identified as the most topologically informative marker (Table 2) across different resources. Analysis using EMTOME revealed significant RNA expression correlations for *EGFR* measured in TGCA cohorts, including BRCA, CHOL (cholangiocarcinoma), and LUAD (lung adenocarcinoma), with limited pathway similarities. For instance, platelet activation in CHOL and EGFR-related signaling in both CHOL and LUAD were highlighted (Supplementary Files S7 and S8). This evidence aligns with the efficacy of anti-*EGFR* treatments in clinical cancer therapies and underscores the need for deeper insight into *EGFR*-signaling pathways in OS as well as in other cancers.

### 3.4. Other Evidence Types

Focusing on GES and trying to reconcile the various differentiated computational frameworks are steps requiring further research, but an even broader global signature space may be explored, including, therefore, other biological/omics layers, such as ncRNAs, methylation, single cells, proteomics, radiomics. To extend this foundation, a few considerations are necessary:

*Integrative signatures: single cells.* Cell-type specific gene expression patterns could reveal prognostic biomarkers for targeted OS treatment. Preliminary single cell analysis has produced a 32-gene meta-signature (e.g., *TPM1*, *S100A13*, *LOXL1*, *PSMD10*, *ST3GAL4*, *PEF1*, *SERPINE2*, *TUBB*, *FAM207A*, *TUBA1A*, *DCN*, *ANXA1*, *TPM1*, *FDPS*, *IFITM5*, *FKBP11*, *SP7*, *SQLE*, *EGFL7*, *VEGFA*, *VEGFB*, *VEGFC*, *VEGFD*, *TNFSF11*, *SFPQ*, *PSPC1*, *NONO*, *NR4A1*, *MUC1*, *COL13A1*, *JAG2*, *KAZALD1*). When mapped onto a STRING.DB interactome measured at the highest confidence level (0.9), the meta-signature delivers an interactome configuration that is very sparse, revealing three little clusters centered on *VEGFA*s studied in the context of angiogenesis dysregulation centered on *EGFL7* [95], on paraspeckles in OS tissue [96] and on a gene set (*TUBA1A*, *TUBB*, *FDPS*, *SQLE*) derived from two prognostic biomarker studies involving osteoclasts [97] and mesenchymal stem cells [98] (Supplementary Files S9a/S9b).

*Integrative omics signatures: ncRNA-omics and radiomics.* An initial screening involving a substantial number of studies showed complex interactions of the ncRNAs landscape, including microRNAs, lncRNAs, pseudogenes etc., and various regulatory mechanisms underlying the observed signatures, such as epigenetics, immune microenvironment, and metabolism, among others. Future work is needed to harmonize evidence across multiple measurements and form aggregates that can be configured as interconnected entities via networks. In principle, a problem may be present regarding the metric to adopt. Similarly, radiomics-linked signatures associating imaging features with genomics (as in radiogenomics, see, for instance, [99,100,101]) may improve outcome prediction but still present challenges in choosing a metric that allows the most effective network integration.

*Further Inferences.* Enhanced inference approaches have focussed on expanding the OS pathogenic genes base and often put emphasis on immune microenvironment-related treatments, such as large-scale fusion of extreme learning machines (ELM), which promises to broaden the OS gene identifications [102]. Our approach contextualizes OS signatures by fusing them within multifaceted metastatic processes and using validated transcriptomic studies. A meta-interactome network can unify knowledge acquired across signatures and apply suitable metrics to define relationships and refine data integration. Representing the signature relationships based on expression profiles provides an initial framework requiring expansion to incorporate other data types. In this context, there is a promising study on gene expression geometrical signatures overperforming standard clustering techniques in Pfizer’s phase II clinical trial data [103].

## 4. Conclusions

Our findings suggest that well-known genes act as universal influencers in OS metastasis, as identified from diverse studies on metabolism and exidative stress. Leveraging a signature assembly is the underlying inference strategy, and we provided examples from recent studies with contextualized analyses that contributed to prioritizing the signature space.

Further refinements may depend on factors worth further investigation that may shape future research directions:

(a) Resolution of signature stratification to account for data heterogeneity;
(b) Interconnectedness across signatures based on selected metrics;
(c) Hypothesis generation power of network configurations to identify critical nodes;
(d) Feasibility of building biologically contextualized networks hierarchically, stepping from single gene/protein to signature, then to signature assembly representing transcriptome sub-profiles, and finally integrations of multiple readouts, experiments, perturbations at various scales;
(e) Combination and harmonization of genetic, molecular, omics, imaging, clinical, and outcome data toward cross-cancer generalizable inferences.

Algorithmically generated signatures carry inherent limitations in causal inference. First, patient stratifications based on shared features may not directly reflect biological mechanisms or therapeutic responses. Additional risk factors for outcome prediction require biological and clinical validations. Second, questionable signature reliability diminishes its interpretative and clinical value, and using surrogate genes as signature components can leave the overall prognostic value unchanged but alter the biological interpretation. Third, reproducibility hinges on rigorous validation across independent patient cohorts, clinical sites, and replicated experiments, free from extraneous model factors.

Our approach can be re-contextualized, i.e., adapted to focus on specific OS mechanisms and processes or generalized to other cancers. Adding complexity from other layers (single cells, epigenetics, ncRNA, imaging, etc.) may necessitate diverse fusion strategies. Ultimately, the integrative network inference approach is adaptable, allowing for perturbation methods, topology variations, metric choice, dynamic analysis to identify genes critical for our understanding of cancer transitions (from normal to tumor states or from non-metastatic to metastatic) and resilience, informing drug robustness and recovery potentials.

## Figures and Tables

**Table 1:** Compendium of GES in osteosarcoma.

**METASTASIS:** *AQP1*, *EVI2B*, *MCAM*, *PLK4*, *A2M*, *MYC*, *IFNGR1*, *MAP3K7*, *LCP2*, *CXCL16*, *CFH*, *GATA3*, *PRC1*, *A2M*
**EMT:** *NID2*, *RPS27A*, *GPX7*, *ITGB5*, *VCAM1*, *FLNA*, *TP53*, *SERPINH1,RB1*
**APOPTOSIS:** *EYA2*, *BAG5*, *IGFBP6*, *IER3*, *TRIM32*, *BNIP3*, *PTPN1*, *PML*, *TERT*, *PSMD10*, *RPS3*, *IFNGR1*, *UBC*,
*UBC*, *HSPB1*, *DNM1L*, *ARHGEF2*, *TGFBR1*
**METABOLISM:** *DDAH2*, *TUBA1A*, *MUC1*, *PEBP1*, *CAT*, *ATG7*
**IMMUNE:** *APOD*, *S100A6*, *FGFRL1*, *BRAF*, *PLD3*, *VEGFA*, *CD79A*, *SDC3*, *GRN*, *RAC3*, *EDNRA*, *VAV1*, *HCK*, *EGFR,*
*PSMC4*, *MYC*, *PPARG*, *CCR5*, *CCL5*
**TME (tumor microenvironment):** *ACTB*, *CSF1R*, *MYC*, *CDK6*, *PML*, *CD4*, *MAP3K5*, *CXCL2*
**AUTOPHAGY:** *BNIP3*, *CCL2*, *VEGFA*, *CALCOCO2*, *EGFR*, *MYC*, *WIPI1*, *DAPK1*
**HYPOXIA:** *EGFR*, *MYH9*, *DCN*, *NDRG1*
**SENESCENCE:** *DHPS*, *BLCAP*, *SUPT5H*, *MYC*, *CAT*, *GRN*, *PTPN1*, *PML*, *UQCRC1*, *MSN*, *TNFRSF10C*, *SLC16A3*, *GLB1*
*CDK6*, *ITGAM*, *MAPK13*, *MAPK14*, *STAT5A*, *STAT5B*, *NOL3*, *G6PD*, *INSR*, *CEBPA*, *MAP3K3*,
*MAP3K5*
**PROGNOSTIC:** *CSNK2B*, *RAD23A*, *USP11*, *MYC*, *MYO6*, *EPB41*, *PML*, *PEF1*

*Note:* “Metastasis” includes signatures distinct from OS metastasis associated with various other processes.

**Table 2:** Top ten NCO and NCE nodes in meta-interactomes. Results from Innate.DB and STRING.DB.

DataBase/Repository/Resource	NODE ID	NAME	DEGREE	BETWEENNESS
***Innate.DB***				
	7316	*UBC*	179	16,123.73
	7137	*TP53*	57	1403.081
	4609	*MYC*	47	588.0141
	1956	*EGFR*	44	595.0013
	2	*A2M*	31	510.33
	60	*ACTB*	29	331.0203
	7412	*VCAM1*	28	174.2803
	5925	*RB1*	27	195.209
	5371	*PML*	26	164.0367
	2316	*FLNA*	26	301.1918
***STRING.DB***				
	7151	*TP53*	69	9558.198
	1956	*EGFR*	67	9017.252
	1432	*MAPK14*	50	5511.467
	7316	*UBC*	45	3530.689
	6233	*RPS27A*	42	2657.348
	4609	*MYC*	39	2565.832
	6776	*STAT5A*	34	1068.389
	6777	*STAT5B*	31	878.6104
	5770	*PTPN1*	31	2078.902
	7409	*VAV1*	30	1488.092

**Table 3:** REACTOME pathway annotations. Sources: Innate.DB and STRING.DB.

Innate.DB Pathway Name	Hits	*p*-Val	*p*-Val (adj.)
*Immune system*	73	2.68E-16	3.76E-13
*Signaling by interleukins*	23	4.88E-15	3.42E-12
*Platelet activation, signaling, and aggregation*	30	1.08E-14	5.06E-12
*Interleukin-3, 5 and GM-CSF signaling*	15	7.29E-13	2.56E-10
*Innate immune system*	42	3.57E-12	1.00E-09
*Hemostasis*	41	8.07E-12	1.89E-09
*Cytokine signaling in immune system*	30	1.19E-11	2.38E-09
*Fcgamma receptor (FCGR) dependent phagocytosis*	17	2.32E-11	4.06E-09
*Adaptive immune system*	46	3.22E-11	4.66E-09
*TCR signaling*	15	3.50E-11	4.66E-09
*Regulation of signaling by CBL*	10	3.65E-11	4.66E-09
*Signaling by the B cell receptor (BCR)*	24	9.37E-11	1.09E-08
*Signalling by NGF*	27	2.16E-09	2.33E-07
**STRING.DB Pathway Name**	**Hits**	***p*-Val**	***p*-Val (adj.)**
*Immune system*	128	1.94E-38	2.72E-35
*Signaling by interleukins*	40	2.26E-29	1.58E-26
*Innate immune system*	76	5.66E-28	2.65E-25
*Platelet activation, signaling, and aggregation*	50	4.23E-27	1.48E-24
*Adaptive immune system*	82	1.11E-25	3.10E-23
*Hemostasis*	72	2.02E-25	4.73E-23
*Signaling by SCF-KIT*	39	1.92E-24	3.85E-22
*Signal transduction*	134	4.70E-24	8.24E-22
*Signalling by NGF*	53	7.06E-24	1.10E-21
*Cytokine Signaling in immune system*	50	1.30E-21	1.83E-19
*Signaling by FGFR in disease*	40	1.69E-21	2.15E-19
*Downstream signal transduction*	38	4.74E-21	5.53E-19
*NGF Signalling* via *TRKA from the plasma membrane*	42	9.79E-21	1.06E-18
*DAP12 signaling*	37	5.50E-20	5.51E-18
*Signaling by the B cell receptor (BCR)*	40	1.31E-19	1.22E-17
*Signaling by FGFR*	36	3.15E-19	2.76E-17
*Signaling by PDGF*	38	1.17E-18	9.65E-17
*Signaling by EGFR in cancer*	37	1.94E-18	1.51E-16
*DAP12 interactions*	37	2.36E-18	1.74E-16
*Signaling by ERBB4*	34	2.72E-18	1.90E-16
*Co-stimulation by the CD28 family*	24	3.43E-18	2.29E-16
*Signaling by ERBB2*	35	4.14E-18	2.61E-16
*GPVI-mediated activation cascade*	18	4.29E-18	2.61E-16
*Signaling by EGFR*	36	1.03E-17	6.04E-16
*Interleukin-3, 5 and GM-CSF signaling*	21	1.11E-17	6.23E-16
*Integrin cell surface interactions*	25	9.82E-17	5.30E-15
*TCR signaling*	22	2.59E-16	1.34E-14
*Downstream signaling of activated FGFR*	31	1.04E-15	5.20E-14
*Interleukin-2 signaling*	18	1.09E-15	5.27E-14
*Fcgamma receptor (FCGR) dependent phagocytosis*	24	1.58E-15	7.39E-14
*Signalling to ERKs*	17	1.64E-15	7.44E-14
*Downstream Signaling Events Of B cell receptor (BCR)*	32	9.90E-15	4.34E-13
*CD28 co-stimulation*	15	1.62E-14	6.87E-13

## Supplementary Materials

The following supporting information can be downloaded at: https://www.mdpi.com/article/10.3390/cancers17010136/s1, Supplementary File S1: GO annotations; Supplementary File S2: regulatory SNAI2; Supplementary File S3: pathway annotations landscape1; Supplementary File S4: annotations landscape2; Supplementary File S5: annotations landscape3; Supplementary File S6: regulatory GATA3; Supplementary File S7: annotations landscape4; Supplementary File S8: annotations landscape5; Supplementary File S9a/S9b: sc-integrated signature/related network.
