# Supplementary material for: Prioritizing Context-Dependent Cancer Gene Signatures in Networks
Source: Cancers (Basel). 2025 Jan 3;17(1):136. doi: 10.3390/cancers17010136 (PMC11720092; doi:10.3390/cancers17010136)
Supplement: Supplementary file 1 [file cancers-17-00136-s001.zip › Suppl File S1.pdf]

Suppl Table S1. GO:BP annotations. Innate.DB and STRING.Db.

GO: BP Name	Innate.DB Hits	P-val	P-val (adj.)
response to wounding	79	4.14E-28	3.39E-25
positive regulation of response to stimulus	83	4.00E-26	1.15E-23
regulation of protein metabolic process	90	4.22E-26	1.15E-23
response to stress	138	9.17E-26	1.88E-23
regulation of immune system process	72	1.95E-25	2.85E-23
regulation of programmed cell death	82	2.08E-25	2.85E-23
regulation of apoptotic process	81	4.90E-25	5.74E-23
intracellular protein kinase cascade	69	1.92E-24	1.97E-22
apoptotic process	93	3.38E-23	2.37E-21
cell activation	93	3.38E-23	2.37E-21
immune system process	62	3.42E-23	2.37E-21
regulation of response to stimulus	106	3.57E-23	2.37E-21
positive regulation of metabolic process	119	3.76E-23	2.37E-21
programmed cell death	105	6.94E-23	4.07E-21
positive regulation of cellular metabolic process	93	8.49E-23	4.64E-21
enzyme linked receptor protein signaling pathway	101	1.32E-22	6.77E-21
intracellular signal transduction	67	3.87E-22	1.87E-20
regulation of cellular protein metabolic process	98	7.99E-22	3.64E-20
response to organic substance	76	3.33E-21	1.44E-19
regulation of defense response	98	3.75E-21	1.54E-19
immune response	44	7.46E-21	2.91E-19
regulation of protein modification process	72	8.20E-21	3.06E-19
wound healing	67	1.07E-20	3.80E-19
regulation of transferase activity	50	1.96E-20	6.71E-19
negative regulation of apoptotic process	52	3.17E-20	9.69E-19
regulation of signal transduction	49	3.30E-20	9.69E-19
positive regulation of immune system process	49	3.30E-20	9.69E-19
regulation of phosphorylation	95	3.37E-20	9.69E-19
negative regulation of programmed cell death	51	3.43E-20	9.69E-19
MAPK cascade	61	4.34E-20	1.19E-18
positive regulation of protein metabolic process	49	6.93E-20	1.83E-18
positive regulation of biological process	48	8.52E-20	2.18E-18
positive regulation of cellular process	61	9.14E-20	2.27E-18
phosphorylation	150	1.26E-19	3.03E-18
response to endogenous stimulus	138	2.05E-19	4.79E-18
hemostasis	76	2.81E-19	6.26E-18
protein phosphorylation	68	2.84E-19	6.26E-18
transmembrane receptor protein tyrosine kinase signaling pathway	44	2.90E-19	6.26E-18
cell proliferation	71	3.53E-19	7.42E-18
regulation of immune response	51	4.01E-19	8.22E-18
response to external stimulus	81	5.00E-19	1.00E-17
blood coagulation	49	5.86E-19	1.14E-17
regulation of body fluid levels	71	8.94E-19	1.70E-17
regulation of molecular function	43	1.26E-18	2.33E-17
coagulation	47	1.28E-18	2.33E-17
regulation of protein phosphorylation	88	1.61E-18	2.86E-17
innate immune response	43	1.64E-18	2.86E-17
positive regulation of signal transduction	56	3.18E-18	5.43E-17
positive regulation of transferase activity	45	3.62E-18	6.06E-17
response to abiotic stimulus	56	5.27E-18	8.64E-17
regulation of cell proliferation	40	8.89E-18	1.43E-16
regulation of catalytic activity	52	9.94E-18	1.57E-16
response to chemical stimulus	67	1.39E-17	2.16E-16
regulation of kinase activity	74	2.80E-17	4.25E-16
defense response	117	4.17E-17	6.21E-16
T cell activation	47	4.34E-17	6.35E-16
regulation of protein kinase activity	68	6.03E-17	8.67E-16
positive regulation of catalytic activity	36	6.94E-17	9.81E-16
positive regulation of cellular protein metabolic process	45	1.12E-16	1.56E-15
positive regulation of protein modification process	56	1.17E-16	1.59E-15
positive regulation of protein phosphorylation	53	1.47E-16	1.97E-15
viral reproductive process	50	1.59E-16	2.10E-15
leukocyte migration	44	2.41E-16	3.13E-15

positive regulation of phosphorylation	41	3.62E-16	4.63E-15
leukocyte activation	30	5.50E-16	6.94E-15
regulation of MAPK cascade	44	5.65E-16	7.02E-15

**GO:BP__** **STRING.DB** **Name___**	**Hits**	**P-val**	**P-val (adj.)**
regulation of protein metabolic process	146	6.83E-56	5.60E-53
protein phosphorylation	132	1.04E-54	4.26E-52
phosphorylation	139	2.02E-54	5.52E-52
regulation of cellular protein metabolic process	134	5.58E-54	1.14E-51
positive regulation of protein metabolic process	114	1.29E-53	2.11E-51
regulation of protein modification process	121	1.90E-53	2.59E-51
intracellular protein kinase cascade	116	2.79E-53	3.26E-51
regulation of phosphorylation	112	2.07E-52	2.12E-50
positive regulation of cellular protein metabolic process	107	5.48E-52	4.99E-50
intracellular signal transduction	161	3.06E-51	2.51E-49
positive regulation of protein modification process	101	6.99E-51	5.21E-49
regulation of protein phosphorylation	106	3.36E-50	2.29E-48
response to stress	204	1.48E-49	9.33E-48
regulation of response to stimulus	184	4.09E-49	2.39E-47
regulation of transferase activity	93	6.42E-48	3.51E-46
positive regulation of phosphorylation	89	1.27E-47	6.51E-46
positive regulation of protein phosphorylation	88	1.77E-47	8.54E-46
regulation of kinase activity	91	3.22E-47	1.47E-45
enzyme linked receptor protein signaling pathway	110	2.50E-46	1.08E-44
positive regulation of response to stimulus	124	1.38E-45	5.67E-44
response to organic substance	155	2.73E-45	1.07E-43
regulation of signal transduction	153	3.08E-45	1.15E-43
regulation of protein kinase activity	86	1.28E-44	4.55E-43
transmembrane receptor protein tyrosine kinase signaling pathway	90	2.30E-44	7.85E-43
regulation of molecular function	146	2.68E-44	8.80E-43
positive regulation of phosphate metabolic process	90	4.86E-44	1.53E-42
positive regulation of metabolic process	159	5.11E-44	1.55E-42
positive regulation of cellular metabolic process	153	3.77E-43	1.10E-41
regulation of catalytic activity	127	8.21E-43	2.32E-41
response to wounding	111	2.06E-42	5.63E-41
positive regulation of signal transduction	97	1.32E-41	3.49E-40
response to chemical stimulus	185	5.06E-41	1.30E-39
MAPK cascade	80	1.65E-40	4.10E-39
protein modification process	170	4.85E-40	1.17E-38
cell surface receptor signaling pathway	171	6.44E-40	1.51E-38
positive regulation of transferase activity	70	5.59E-39	1.27E-37
response to endogenous stimulus	107	1.27E-37	2.81E-36
macromolecule modification	170	1.42E-37	3.06E-36
regulation of MAPK cascade	71	2.78E-37	5.85E-36
positive regulation of catalytic activity	94	1.55E-36	3.18E-35
wound healing	77	4.06E-36	8.13E-35
regulation of immune system process	98	9.77E-36	1.91E-34
regulation of programmed cell death	110	1.11E-34	2.11E-33
positive regulation of cellular process	197	2.19E-34	4.09E-33
programmed cell death	129	4.59E-34	8.36E-33
apoptotic process	127	2.83E-33	4.94E-32
signal transduction	127	2.83E-33	4.94E-32
regulation of apoptotic process	226	3.09E-33	5.27E-32
cell activation	107	5.77E-33	9.66E-32
response to external stimulus	85	6.10E-33	1.00E-31
cell proliferation	106	7.03E-33	1.13E-31
regulation of body fluid levels	119	1.07E-32	1.69E-31
blood coagulation	72	1.47E-32	2.27E-31
coagulation	66	2.32E-32	3.53E-31
negative regulation of programmed cell death	66	3.58E-32	5.33E-31
hemostasis	72	4.18E-32	6.13E-31
interaction with host	66	4.43E-32	6.38E-31
immune system process	58	6.41E-32	9.06E-31
negative regulation of apoptotic process	142	9.28E-32	1.29E-30
viral reproductive process	71	9.78E-32	1.31E-30
cell migration	71	9.78E-32	1.31E-30

regulation of immune response	66	7.33E-31	9.69E-30
