# Supplementary material for: Prioritizing Context-Dependent Cancer Gene Signatures in Networks
Source: Cancers (Basel). 2025 Jan 3;17(1):136. doi: 10.3390/cancers17010136 (PMC11720092; doi:10.3390/cancers17010136)
Supplement: Supplementary file 1 [file cancers-17-00136-s001.zip › Suppl File S2.pdf]

Suppl Table S2. Regulatory relationships involving SNAI2. TF regulators (left-most column) and SNAI2-regulated targets (top panel), together with shared co-regulated targets (bottom panel). TRRUST db source (including mode of regulation: activation or repression extracted from thousands currently known).

**TFs that regulate SNAI2**			**SNAI2 targets**			
**TF**	**Target**	**Regulation Mode**		**TF**	**Target**	**Regulation Mode**
	**SNAI2**			**SNAI2**		
CREB1		Repression			*CD44*	Activation
ESRRA		Repression			*CDH1*	Repression
EZH2		Repression			*CDH1*	Unknown
HDAC1		Repression			*CDH2*	Unknown
HDAC2		Repression			*CXADR*	Repression
LEF1		Unknown			*CXCL12*	Activation
MTA1		Activation			*CXCR4*	Activation
NCOR1		Repression			*HPGD*	Repression
PER2		Repression			*JAG1*	Activation
POU2F1		Activation			*LPAR2*	Repression
POU2F1		Repression			*MMP17*	Unknown
RUNX2		Activation			*MMP9*	Activation
SUZ12		Repression			*POU5F1*	Activation
TCF7		Unknown			*UBE2D3*	Repression
TCF7L2		Unknown			*VDR*	Repression
WT1		Unknown				

**TFs that share targets with SNAI2**	***P values from hypergeometric test.**			
**TF**		**# of overlapping target genes**		
			**P value**	**FDR**
SNAI1		4	2.37E-10	3.01E-09
HIF1A		6	3.86E-10	4.71E-09
SP1		9	1.11E-09	1.15E-08
TWIST2		4	1.41E-08	1.08E-07
ZEB2		3	2.37E-08	1.68E-07
YBX1		4	3.50E-08	2.36E-07
TWIST1		4	6.53E-08	4.06E-07
KLF8		3	9.94E-08	5.78E-07
ZEB1		3	4.29E-07	2.06E-06
STAT3		5	5.83E-07	2.67E-06
JUN		5	6.42E-07	2.90E-06
TFAP2A		4	1.05E-06	4.38E-06
HDAC1		4	1.70E-06	6.52E-06
HDAC3		3	3.04E-06	1.05E-05
ETS1		4	3.11E-06	1.07E-05

SRF		3	5.24E-06	1.65E-05
KLF4		3	1.06E-05	2.95E-05
EZH2		3	1.23E-05	3.34E-05
IKBKB		2	1.77E-05	4.46E-05
MZF1		2	1.77E-05	4.46E-05
PAX3		2	1.77E-05	4.46E-05
RELA		5	1.99E-05	4.91E-05
NFKB1		5	2.02E-05	4.97E-05
SALL4		2	3.29E-05	7.38E-05
WT1		3	3.35E-05	7.47E-05
SMARCA4		2	4.23E-05	9.00E-05
SPDEF		2	6.45E-05	1.26E-04
PAX6		2	7.74E-05	1.47E-04
TCF3		2	1.23E-04	2.15E-04
CREB1		3	1.36E-04	2.33E-04
HMGA1		2	1.79E-04	2.92E-04
KLF5		2	2.22E-04	3.49E-04
KLF6		2	2.22E-04	3.49E-04
RUNX2		2	2.22E-04	3.49E-04
SP3		3	2.85E-04	4.32E-04
ETV4		2	3.21E-04	4.79E-04
MTA1		2	3.77E-04	5.46E-04
ERG		2	4.38E-04	6.20E-04
RUNX3		2	5.04E-04	6.94E-04
DNMT1		2	5.38E-04	7.35E-04
SMAD3		2	5.74E-04	7.76E-04
ETS2		2	6.87E-04	9.00E-04
SIRT1		2	1.69E-03	1.98E-03
PPARG		2	2.65E-03	2.97E-03
ESR1		2	3.28E-03	3.60E-03
STAT1		2	4.75E-03	5.07E-03
AR		2	5.69E-03	6.01E-03
TP53		2	1.45E-02	1.47E-02
