# Supplementary material for: Prioritizing Context-Dependent Cancer Gene Signatures in Networks
Source: Cancers (Basel). 2025 Jan 3;17(1):136. doi: 10.3390/cancers17010136 (PMC11720092; doi:10.3390/cancers17010136)
Supplement: Supplementary file 1 [file cancers-17-00136-s001.zip › Suppl File S6.pdf]

Suppl Table S3. Regulatory relationships involving GATA3. GATA3 regulators, regulated targets and shared targets with other TFs from TRRUST db.

**GATA3 targets**			**TFs that share targets with GATA3**			
**TF**	**Target**	**Mode of Regulation**	**P values are calculated with hypergeometric test.*			
**GATA3**			**TF**	**# overlapping target genes**	**P value**	**FDR**
	CD40LG	Activation				
	CD8A	Activation	*TBX21*	7	1.02E-15	3.59E-14
	CD8B	Unknown	*SP1*	15	6.93E-14	1.84E-12
	CDH1	Activation	*PARP1*	7	1.83E-13	4.53E-12
	CDH3	Repression	*TAL1*	5	1.07E-11	1.85E-10
	CDX2	Activation	*RELA*	11	5.96E-11	8.80E-10
	CHST4	Activation	*NFKB1*	11	6.17E-11	9.05E-10
	CXCL1	Repression	*EP300*	7	9.06E-11	1.28E-09
	EPO	Repression	*AR*	7	2.53E-09	2.38E-08
	EPOR	Unknown	*HOXA9*	4	2.65E-09	2.49E-08
	ERG	Activation	*BRCA1*	6	3.46E-09	3.13E-08
	ERVW-1	Activation	*POU2F1*	5	2.03E-08	1.48E-07
	ESR1	Unknown	*STAT5A*	4	2.09E-08	1.51E-07
	FOXC1	Repression	*SATB1*	4	2.85E-08	1.97E-07
	FOXC2	Repression	*FHL2*	3	1.02E-07	5.91E-07
	FUT7	Unknown	*PGR*	4	4.18E-07	2.02E-06
	HSD17B1	Repression	*STAT5B*	3	5.70E-07	2.62E-06
	IFNG	Unknown	*STAT3*	6	1.02E-06	4.29E-06
	IL10	Unknown	*LMO2*	3	1.22E-06	4.93E-06
	IL12RB2	Repression	*HIF1A*	5	1.30E-06	5.23E-06
	IL4	Unknown	*LEF1*	4	1.33E-06	5.31E-06
	IL5	Activation	*NFATC2*	3	1.67E-06	6.42E-06
	IL5	Unknown	*TP53*	6	1.71E-06	6.56E-06
	KLK1	Unknown	*ETS1*	5	1.92E-06	7.26E-06
	LOX	Repression	*FOS*	4	7.69E-06	2.27E-05
	MMP1	Unknown	*GATA1*	4	8.84E-06	2.54E-05
	MUC1	Activation	*NFKBIA*	3	9.70E-06	2.74E-05
	NFKB1	Unknown	*CEBPB*	4	1.22E-05	3.32E-05
	PAEP	Activation	*PAX5*	3	1.33E-05	3.52E-05
	RELA	Unknown	*HDAC9*	3	1.53E-05	3.97E-05
	SLCO4C1	Unknown	*FLI1*	3	2.28E-05	5.49E-05
	STAT4	Repression	*ESR1*	4	2.55E-05	6.03E-05
	TCF7L2	Unknown	*NR1I2*	3	2.57E-05	6.08E-05
	TEK	Activation	*HDAC1*	4	2.97E-05	6.77E-05
	TGFBR3	Unknown	*BCL3*	2	4.82E-05	9.87E-05
	TRA	Unknown	*KDM4B*	2	4.82E-05	9.87E-05

	ZEB1	Activation		*PIAS2*	2		4.82E-05	9.87E-05
				PIAS3	2		4.82E-05	9.87E-05
**TFs that regulate GATA3**				*TNFAIP3*	2		4.82E-05	9.87E-05
**TF**	**Target**	**Mode of Regulation**		*ATF2*	3		5.34E-05	1.08E-04
	**GATA3**			*STAT1*	4		5.37E-05	1.08E-04
MEN1		Unknown		*JUND*	3		5.85E-05	1.16E-04
MYB		Activation		*ETS2*	3		6.39E-05	1.25E-04
NFKB1		Activation		*TWIST1*	3		6.96E-05	1.34E-04
RELA		Activation		*KLF4*	3		8.87E-05	1.64E-04
SATB1		Unknown		*EZH2*	3		1.03E-04	1.86E-04
SP1		Unknown		*APEX1*	2		1.34E-04	2.31E-04
TBX21		Unknown		*MAF*	2		1.34E-04	2.31E-04
TCF7L2		Unknown		*SALL4*	2		1.34E-04	2.31E-04
				SMARCA4	2		1.72E-04	2.85E-04
				NKX3-1	2		2.15E-04	3.42E-04
				IRF4	2		2.63E-04	4.03E-04
				MECP2	2		2.63E-04	4.03E-04
				MSC	2		2.63E-04	4.03E-04
				POU2F2	2		3.15E-04	4.71E-04
				USF1	3		4.26E-04	6.05E-04
				ZEB1	2		4.33E-04	6.13E-04
				FOXA2	2		7.24E-04	9.42E-04
				GLI2	2		7.24E-04	9.42E-04
				HMGA1	2		7.24E-04	9.42E-04
				GATA2	2		8.96E-04	1.13E-03
				KLF6	2		8.96E-04	1.13E-03
				NR1H4	2		8.96E-04	1.13E-03
				ARNT	2		1.09E-03	1.34E-03
				REL	2		1.09E-03	1.34E-03
				EGR1	3		1.09E-03	1.34E-03
				YY1	3		1.27E-03	1.53E-03
				HDAC4	2		1.29E-03	1.56E-03
				NANOG	2		1.41E-03	1.68E-03
				MTA1	2		1.52E-03	1.80E-03
				BCL6	2		1.76E-03	2.05E-03
				ERG	2		1.76E-03	2.05E-03
				HDAC2	2		1.76E-03	2.05E-03
				DNMT1	2		2.16E-03	2.46E-03
				YBX1	2		2.16E-03	2.46E-03
				SP3	3		2.24E-03	2.55E-03
				HSF1	2		2.45E-03	2.76E-03
				NR3C1	2		3.23E-03	3.55E-03

				RUNX1	2			3.76E-03	4.07E-03
				JUN	3			4.94E-03	5.26E-03
				VDR	2			5.33E-03	5.66E-03
				IRF1	2			5.98E-03	6.30E-03
				CEBPA	2			6.67E-03	6.99E-03
				SIRT1	2			6.67E-03	6.99E-03
				WT1	2			7.15E-03	7.47E-03
				PPARG	2			1.03E-02	1.06E-02
				TFAP2A	2			1.09E-02	1.12E-02
				CREB1	2			1.75E-02	1.77E-02
				MYC	2			2.45E-02	2.47E-02
				E2F1	2			3.93E-02	3.94E-02
