# Supplementary figures and images for: Prioritizing Context-Dependent Cancer Gene Signatures in Networks
Source: Cancers (Basel). 2025 Jan 3;17(1):136. doi: 10.3390/cancers17010136 (PMC11720092; doi:10.3390/cancers17010136)

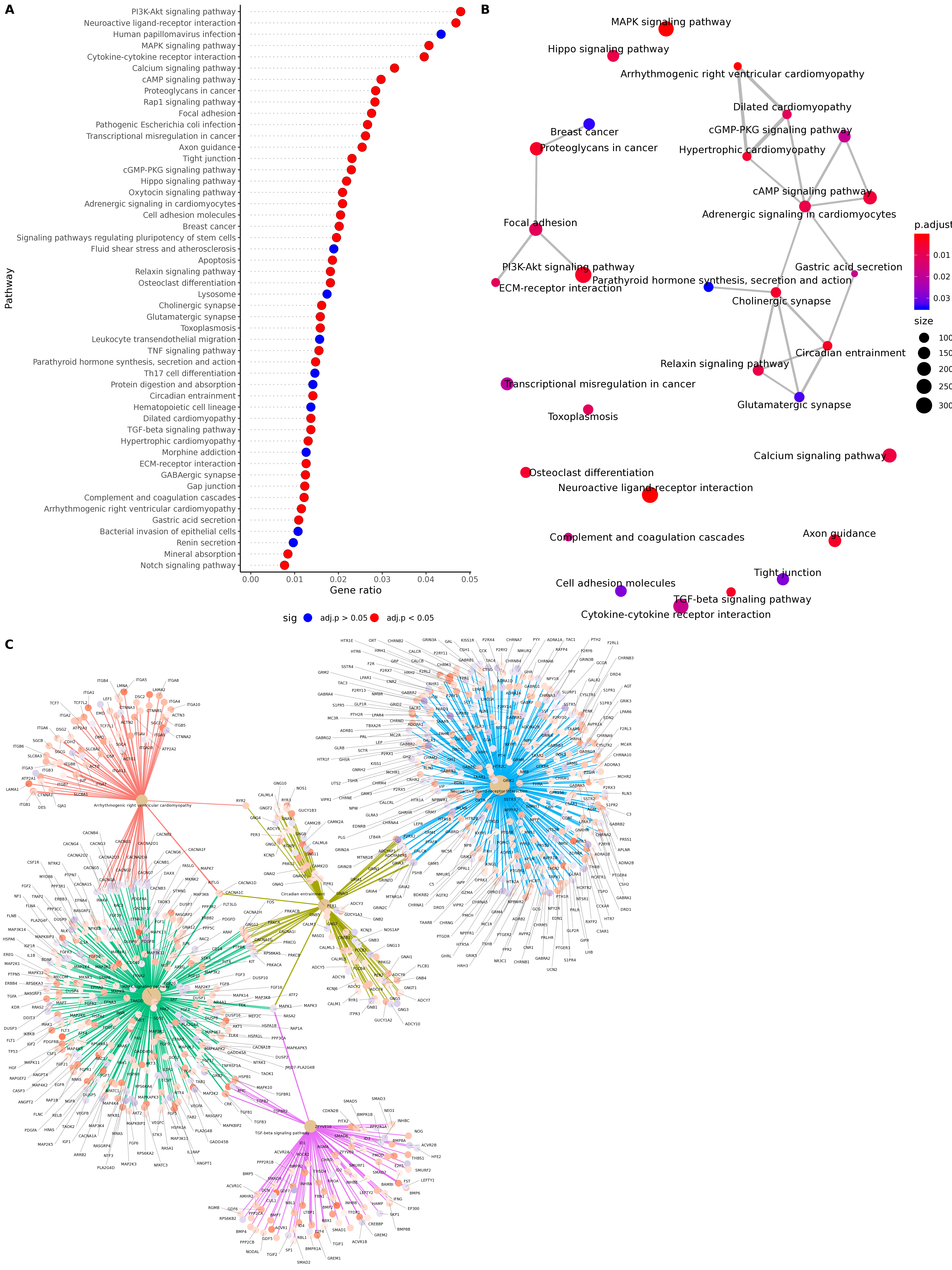

Supplement: Supplementary file 1 [file cancers-17-00136-s001.zip › Suppl File S3.png]

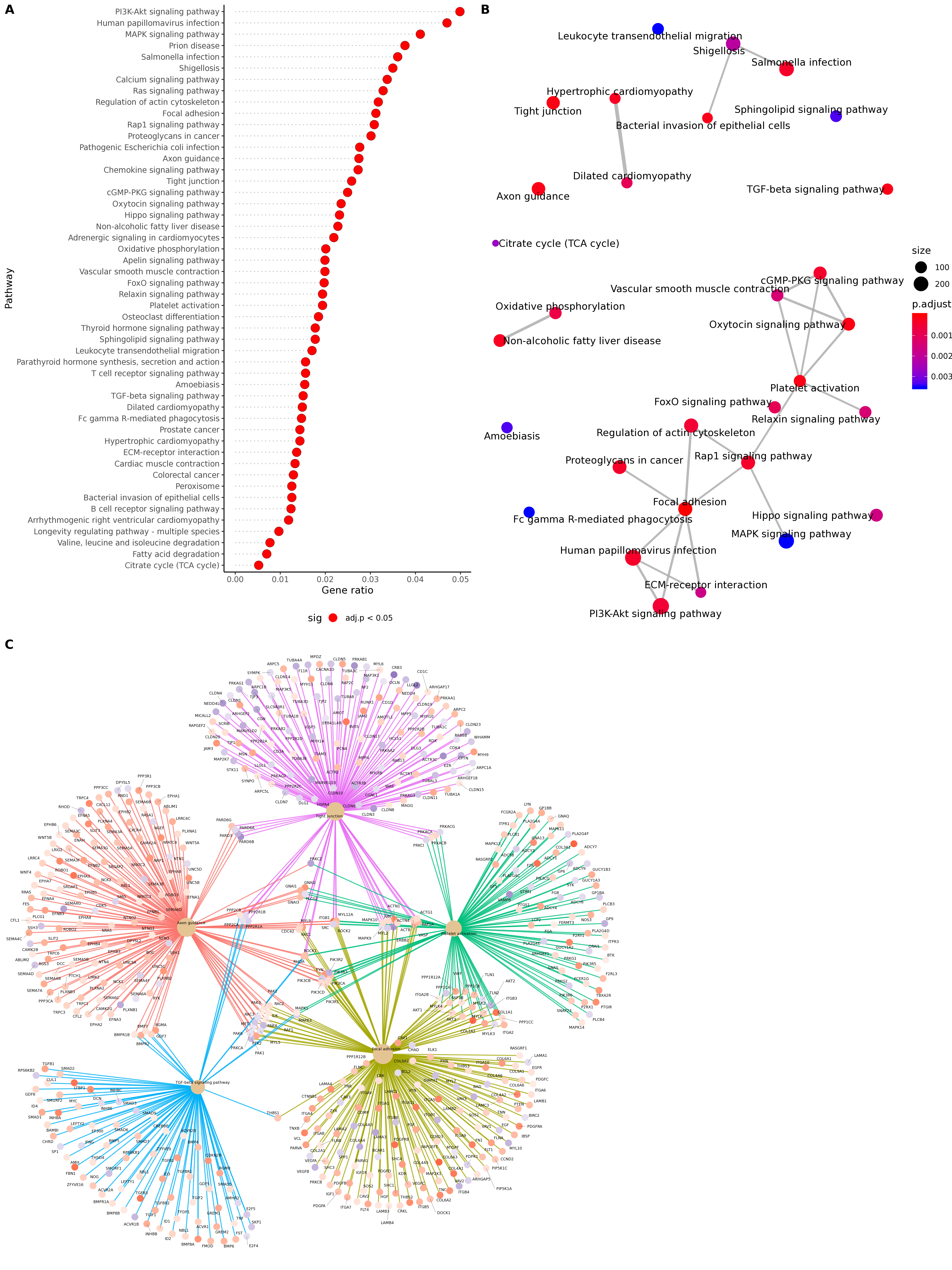

Supplement: Supplementary file 1 [file cancers-17-00136-s001.zip › Suppl File S4.png]

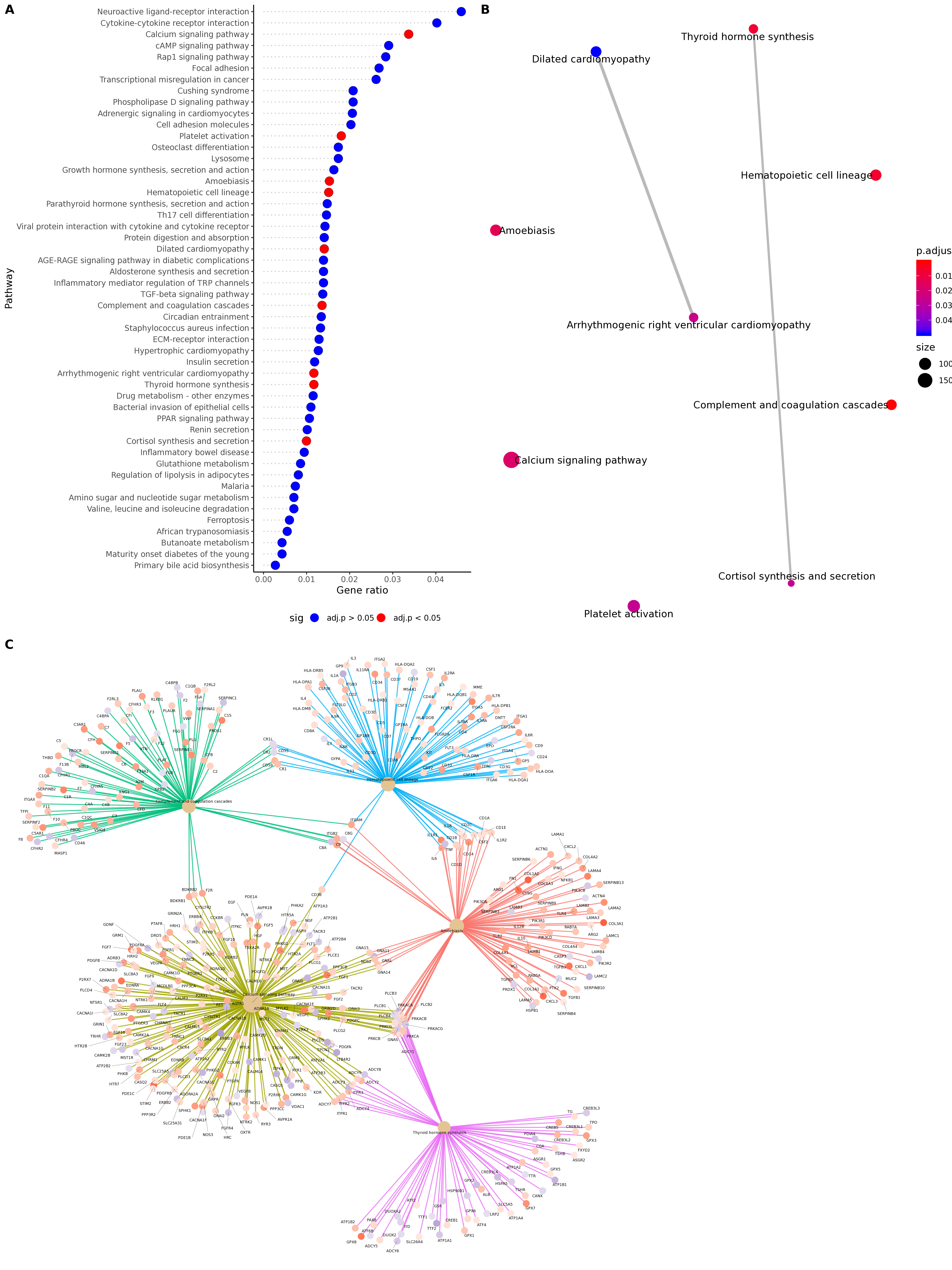

Supplement: Supplementary file 1 [file cancers-17-00136-s001.zip › Suppl File S5.png]

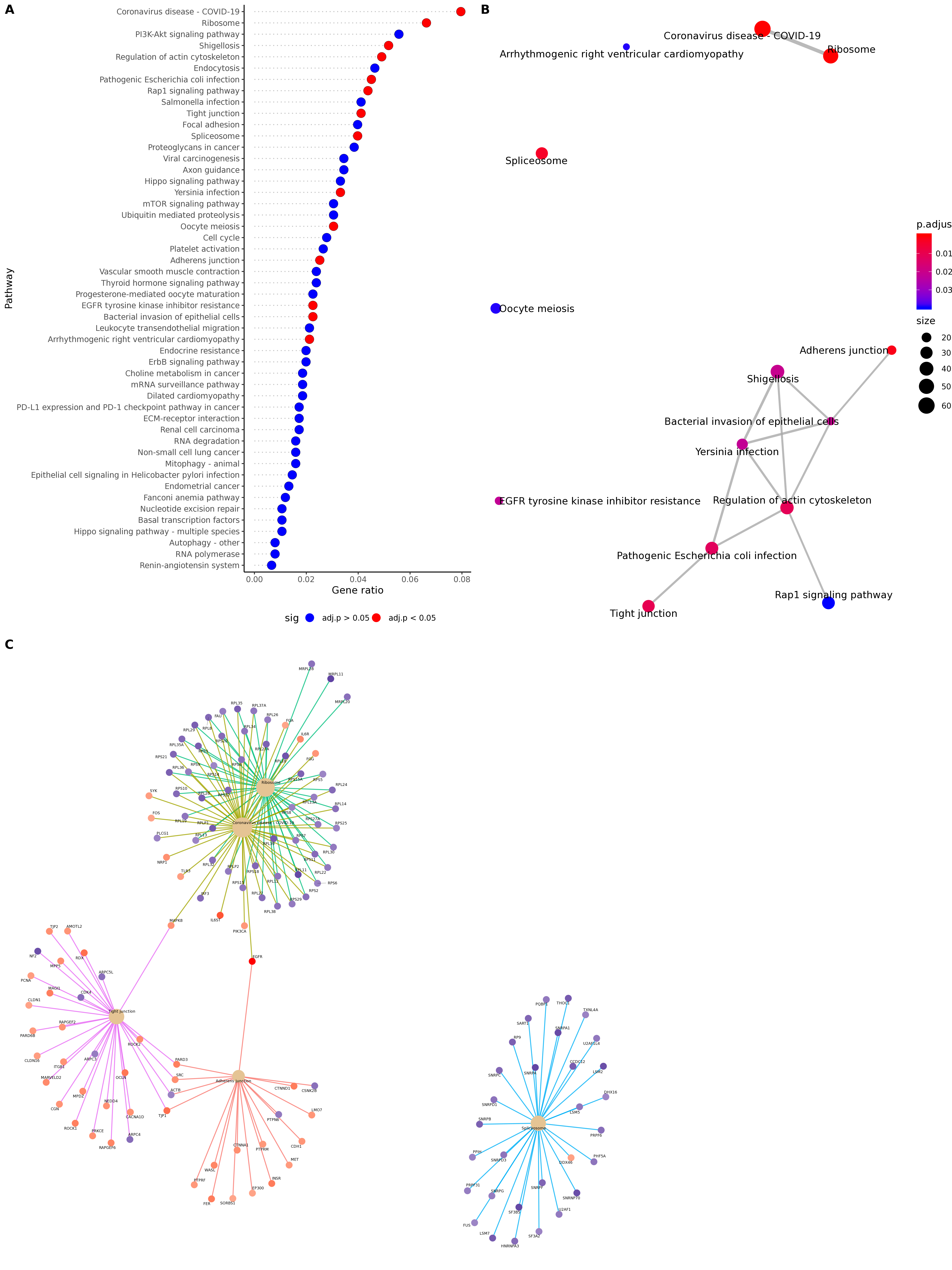

Supplement: Supplementary file 1 [file cancers-17-00136-s001.zip › Suppl File S7.png]

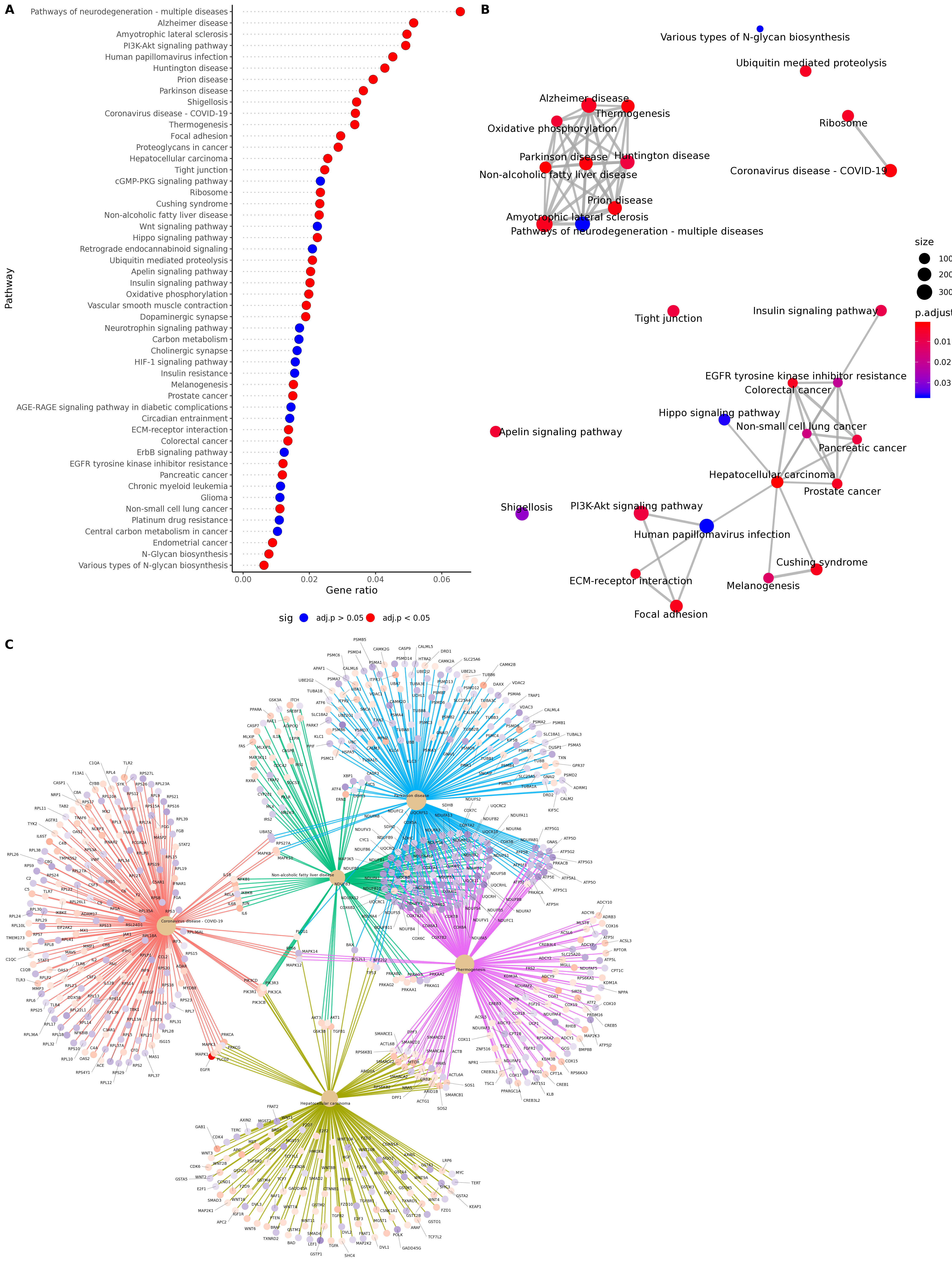

Supplement: Supplementary file 1 [file cancers-17-00136-s001.zip › Suppl File S8.png]

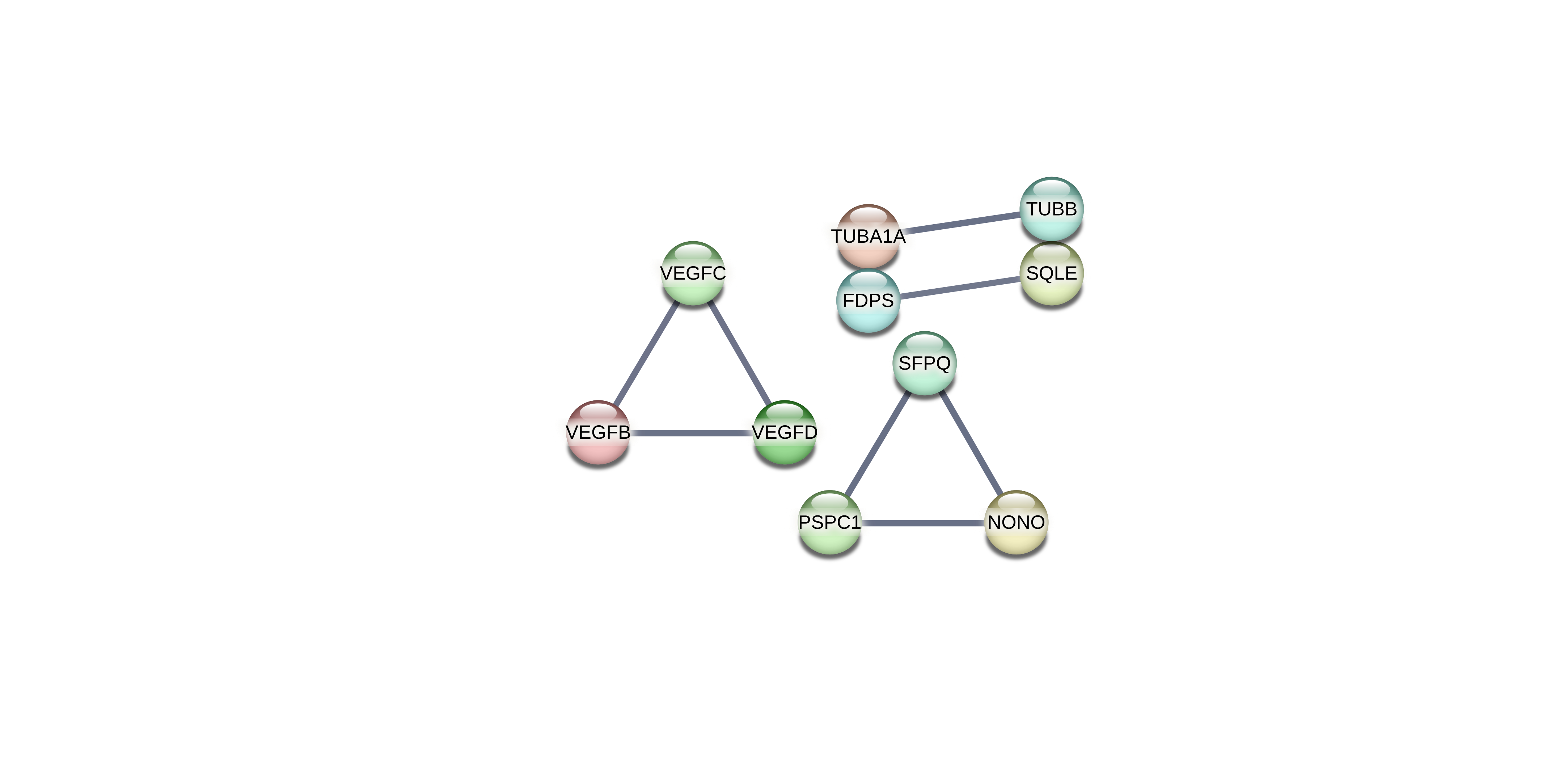

Supplement: Supplementary file 1 [file cancers-17-00136-s001.zip › Suppl File S9b.png]
